# National Insurance Act Developments

**Published:** 1915-06-05

**Authors:** 


### NATIONAL INSURANCE ACT DEVELOPMENTS.

### The Decision of Disputes.

The Insurance Commissioners have recently published a book in which particulars are given of decisions on appeals made under the provisions of the National Insurance Acts in cases of dispute. The object of this publication is to provide information on the subject for the use of approved societies generally and for others interested in the administration of the Acts. The actual decisions are in themselves of some importance, but the principal value of the publication lies in the fact that the method of dealing with the cases in question is clearly set forth, so that it is possible for others to obtain an exact idea of the procedure involved in the decision of disputes as they arise. It was considered advisable when the National Insurance scheme was under dicussion to incorporate in the Act the necessary provision for the decision of disputes without making it necessary to apply to the Courts. This was in fact what was actually done; but, in applying the powers conferred upon them, the Insurance Commissioners have thought fit to require such ample investigation on the lines of the normal procedure in the taking of evidence that it is doubtful whether any great saving has been made either in time or money through dispensing with the ordinary right of application to the Courts. We have recently heard of cases where approved societies have disputed claims on the ground of malingering, and although the disputes have been settled in their favour the procedure has been so costly that there is every reason to shrink from taking further action of a similar character. This is a very undesirable state of affairs, and demands the serious attention of the Insurance Commissioners, for it is clear that if approved societies are willing to pay claims in cases where there is doubt as to their validity rather than take the risk of incurring heavy expenses, even though the decision should be in their favour, the cost of these doubtful claims must fall upon the general body of the members and upon the community at large.

### The Effect of the War on Administration Expenses of Approved Societies.

By the transfer of so many members from the ordinary class of employed contributors to the Navy and Army class, owing to the members having joined the Forces, large numbers of approved societies were in difficulties in regard to their administration expense accounts. It is true that the societies are relieved of the expense of administering sickness and disablement benefits in respect of such transferred members; but, in view of the fact that the members in question are amongst the most healthy while those suffering from sickness and incapacity remain, the societies have not the full advantage of the relief which such transference would at first sight appear to give. Moreover, the clerical labour involved in the work of registering the transfers is considerable, and the societies have had, in addition, to face the question of revising the terms upon which their representatives—secretaries or agents—are remunerated.

In view of these facts it has now been decided by the Insurance Commissioners to allow the approved societies a maximum sum of 1s. 8½d. for each half-year in which a transfer is made from the employed contributor class to the Navy and Army class, or *vice versa*; and to allow 10¼d. instead of 4d. for each of the half-years intervening.

